# Vildagliptin Reduces Stenosis of Injured Carotid Artery in Diabetic Mouse Through Inhibiting Vascular Smooth Muscle Cell Proliferation via ER Stress/NF-κB Pathway

**DOI:** 10.3389/fphar.2019.00142

**Published:** 2019-02-25

**Authors:** Yuqin Ji, Yingbin Ge, Xinyu Xu, Sen Ye, Yuansheng Fan, Jing Zhang, Lianlian Mei, Xiaofeng Zhang, Lianghong Ying, Tao Yang, Chunjian Li

**Affiliations:** ^1^First Clinical Medical School, Nanjing Medical University, Nanjing, China; ^2^Department of Physiology, Nanjing Medical University, Nanjing, China; ^3^Department of Endocrinology, The First Affiliated Hospital of Nanjing Medical University, Nanjing, China; ^4^Department of Cardiology, The Second Affiliated Hospital of Medical School of Southeast University, Nanjing, China; ^5^Department of Cardiology, The Affiliated Huai’an Hospital of Xuzhou Medical University, Huai’an, China; ^6^Department of Cardiology, The First Affiliated Hospital of Nanjing Medical University, Nanjing, China

**Keywords:** vildagliptin, VSMCs proliferation, phospho-IRE-1, phospho-p65, phospho-IKKα/β

## Abstract

Dipeptidyl peptidase-4 (DPP-4) inhibitors are novel anti-hyperglycemic drugs for type 2 diabetes. It has been reported that DDP-4 inhibitor could exert pleiotropic effects on cardiovascular system. This study was to explore the effect and mechanism of vildagliptin on the stenosis of injured carotid artery in diabetic mouse. Twenty six-week-old male db/db mice (BKS) were randomized into vildagliptin treated and vehicle control groups. Ligation injury was first performed in left carotid arteries of all diabetic mice, then oral vildagliptin or equal amount of PBS was correspondingly administered to the mice from the next day to ligation injury for 4 weeks. Effects on proliferation were detected via histological and morphometric analysis. Endoplasmic reticulum (ER) stress and nuclear factor kappa B (NF-κB) markers were determined by immunoblot analysis. After 4 weeks of vildagliptin delivery, it was observed that the intimal area and neointimal thickness of the ligated carotid arteries were significantly reduced as compared to the control group. *In vivo*, vildagliptin suppressed the expressions of PCNA and α-SMA, phospho-p65, phospho-IKKα/β, GRP78 and CHOP, as well as IRE-1 in vascular smooth muscle cells (VSMCs). *In vitro*, the proliferation and hypertrophy of VSMCs were significantly inhibited by blocking the IRE-1 pathway, and the inhibition of phospho-IRE-1 expression down-regulated the expression of phospho-IKKα/β in VSMCs. Vildagliptin reduced the stenosis of injured carotid arteries in diabetic mice, and this effect was achieved via inhibiting the activation of ER stress/NF-κB pathway.

## Introduction

Percutaneous coronary intervention (PCI), as a therapeutic strategy for coronary artery diseases, is now widely applied in clinic (Kim et al., 2008). However, the high incidence of in-stent restenosis subsequent to PCI and the lack of valid medical therapeutic measures have become an urgent issue to be resolved. Studies suggested a tendency that number of PCI cases increased in individuals complicated with type-2 diabetes mellitus, and such particular patients suffered a higher risk of in-stent restenosis compared with non-diabetic patients (Rodriguez et al., 2007; Lu et al., 2017). Therefore, further study in identifying mechanism and intervention of restenosis for diabetic patients is of great significance.

It is well known that type-2 diabetes is a metabolic disorder characterized by hyperglycemia, which simultaneously results in chronic non-communicable inflammation in artery and heart (Gaede et al., 2008). Besides, hyperglycemia serves as a primary factor in stimulating proliferation and hypertrophy of VSMCs (Werstuck et al., 2006). Nevertheless, it is uncertain whether restenosis from diabetic patients is induced by hyperglycemia and related to proliferation and hypertrophy of VSMCs; and the involved mechanism remains unclear.

Dipeptidyl peptidase-4 (DPP-4) inhibitors are currently utilized to lower blood glucose via increasing the concentration of Glucagon-like peptide (GLP-1), which is produced by L-type cells in the intestine and serves as anti-hyperglycemic endogenous hormone (Drucker, 1998; Astrup et al., 2009; Neumiller et al., 2010). Recent studies reported that DPP-4 inhibitors including sitagliptin and alogliptin exerted pleiotropic effects in modulating cardiovascular disorders, in which sitagliptin attenuated intimal hyperplasia in response to vascular injury in rats; alogliptin reduced atherosclerotic lesions in a mouse model (Shah et al., 2011; Matsubara et al., 2012; Ishii et al., 2014); however, to the best of our knowledge, whether the latest DPP-4 inhibitors, vildagliptin, has any effect on the injury induced intimal hyperplasia has not yet been investigated.

In this study, we constructed a carotid artery stenosis model in type-2 diabetic mice to investigate the anti-proliferative and anti-hypertrophic effects of vildagliptin on VSMCs in the carotid artery and the associated mechanisms, by which we aimed to explore an effective method to treat the in-stent restenosis after PCI.

## Materials and Methods

### Animals

Type-2 diabetic mouse model (db/db) was used in this study. Twenty six-week-old male db/db mice (BKS) were purchased from Model Animal Research Center of Nanjing University (Nanjing, China). All animal experiments were complied with rules of the NIH Guide for the Care and Use of Laboratory Animals. This study was approved by the Animal Ethical and Welfare Committee of Nanjing Medical University (Permit Number: IACUC-1601027). All mice were group housed on a 12 h light/dark cycle at 23°C, with free access to food and water.

### Carotid Restenosis Model and Vildagliptin Treatment

After 1 week of acclimation, twenty mice were randomized into vildagliptin treated and vehicle control groups, and anesthetized with an intraperitoneal injection of ketamine (35 mg/kg) and xylazine (5 mg/kg) and then subjected to ligation injury as previously described (Yoshida et al., 2008). Briefly, the left common carotid artery was located and ligated using a 5–0 silk suture just proximal to the bifurcation. Vildagliptin (Novartis Pharma AG, Switzerland) was administered 35 mg/kg daily by oral gavage to the 10 mice (which was equal to 3.85 mg/kg daily for humans) in the vildagliptin treated group for 4 weeks. Another 10 mice in the vehicle control group were administered equal amount of PBS for 4 weeks. At the end of experiment, mice were culled and both of the left ligated carotid arteries and the right un-ligated carotid arteries (sham operation control) were collected for histological or biochemical analysis.

### Body Weight and Blood Glucose

Body weight and non-fasting blood glucose were measured once a week throughout the entire course of the experiment. Blood samples were obtained from jugular veins. Non-fasting blood glucose was monitored by a MEDISAFE Blood Glucose Test TIP (MS-GC308, Terumo Corporation).

### Histological and Morphometric Analysis

Carotid arteries were perfused and fixed with 4% paraformaldehyde and embedded in paraffin. Cross-sections (5 μm) were cut and stained with hematoxylin and eosin. Lumen areas were measured using Image-Pro Plus software (Media Cybernetics Silver Spring, MD) as previously described (Zhang et al., 2015).

### Immunohistochemistry and Immunofluorescence

For immunohistochemistry, paraffin sections were deparaffinized and rehydrated. Antigen retrieval was performed with the citrate buffer (pH 6.0) by heating the slides for 15 min, slides were sequentially incubated with 3% H_2_O_2_ and blocking buffer. Sections were incubated overnight at 4°C with the primary antibodies (described as follows). Two-step technique (SuperPicture^TM^ 3rd Gen IHC Detection kit; Invitrogen, Carlsbad, CA, United States) was used for visualization, with DAB for color development. Finally, sections were counterstained with hematoxylin and mounted. Primary antibodies for immunocytochemistry were used at the following dilutions: rabbit anti-α-smooth muscle actin (1:500, Abcam, Cambridge, MA, United States), mouse anti-PCNA (1:200, Servicebio, Wuhan, China), goat anti-CD31 (1:200, Servicebio, Wuhan, China). For immunofluorescence, sections were incubated overnight at 4°C with a mixture of rabbit anti-α-smooth muscle actin (1:500, Abcam, Cambridge, MA, United States) and mouse anti-PCNA (1:200, Servicebio, Wuhan, China). The sections were then treated with a mixture of Alexa 568-conjugated goat anti-mouse IgG (1:500; Jackson ImmunoResearch Labs, United States) and Alexa 488-conjugated goat anti-rabbit IgG (1:1000, Jackson ImmunoResearch Labs, United States) for 1 h at room temperature. Imaging was acquired on an Olympus BX51 microscope using an Olympus DP70 digital camera, and photographs of the tissue specimens were taken at 100× magnification. Negative controls had the primary antibody omitted or replaced by non-immune as the primary antibodies.

### Western Blotting

Total proteins in carotid arteries and cells were extracted by RIPA buffer according to the manufacturer’s instructions (Beyotime Biotechnology, Shanghai, China) and quantified by BCA Protein Assay Kit (Pierce, Rockford, IL, United States). Equal amount proteins were separated by 10% SDS-PAGE and transferred to PVDF membranes (Millipore, Bedford, MA, United States). The membranes were blocked with 5% non-fat milk and then incubated with primary antibodies (described as follows) at 4°C overnight, followed by detection with horseradish peroxidase-conjugated secondary antibodies (1:5000, Santa Cruz Biotechnology, Santa Cruz, CA, United States). An ECL detection kit (Tanon Biotechnology, Shanghai, China) was used to detect protein expression levels. Primary antibodies were used at the following dilutions: PCNA [1:1000, (1:200, Servicebio, Wuhan, China)], anti-α-smooth muscle actin (1:1000, Abcam, Cambridge, MA, United States), Cyclin D, CDK2 (1:1000, Santa Cruz Biotechnology, Santa Cruz, CA, United States), phospho-p65, p65 (1:1000, Abcam, Cambridge, MA, United States), phospho-IKKα/β, IKKα, IKKβ (1:1000, Cell Signaling Technology, Danvers, MA, United States), TNF-α, IL-1β (1:1000, GeneTex, Irvine, CA, United States), CHOP, GRP78 (1:1000, Proteintech, CHI, United States), ATF6, phospho-eIF2α, eIF2α, phospho-IRE-1, IRE-1, (1:1000, Cell Signaling Technology, Danvers, MA, United States). Signal intensity was quantified using fluorography, as enhanced by the electrochemiluminescence system (WBKLS0100 from Millipore, MA, United States). The results were normalized using GAPDH as internal control. For background, the primary antibody was omitted.

### Cell Culture and Treatment

The rat thoracic aortic smooth muscle cell line A7r5 (#BNCC339542) was purchased from the cell bank of the Chinese Academy of Sciences, Shanghai, China. A7r5 cells were cultured in Dulbecco’s Modified Eagle’s Medium (DMEM) (11995065/11885092 from Gibco, CA, United States), which was supplemented with 100 U/mL penicillin, 100 μg/mL streptomycin (450-201-EL from Wisent Inc., CA, United States), and 10% FBS (10099-141 from Gibco, CA, United States), after which the cells were seeded at 1.25 × 104 per well in 6-well culture plates and allowed to grow for 2 days in DMEM, then the cells were divided into four groups and treated with DMEM solution containing 5.5 mM glucose (Normal glucose), 33 mM glucose (High glucose), 33 mM glucose + ACHP (High glucose + ACHP) or 33 mM glucose + turine (High glucose + taurine), respectively (Wang K. et al., 2017), where ACHP was added at a final concentration of 10 μM (Ma et al., 2015) and taurine at a final concentration of 25 mM (Pan et al., 2010). In all experiments, cells were serum-starved for 12 h with DMEM containing 0.5% FBS and then subjected to the different treatments.

### Cell Counting Kit-8 and EdU Incorporation Assay

Cell counting kit-8 (CCK-8) assay (Dojindo, Kumamto, Japan) and EdU (5-ethynyl-2- deoxyuridine) incorporation assay were adopted to evaluate the cell proliferation process. After the A7r5 cells were seeded and treated as previous described, we subsequently added CCK-8 solution (10 μL) to each well, and the wells were incubated for a further 2 h at 37°C. The absorbance was then measured at 450 nm (BioTek, Elx800, United States). A Click-iTH EdU Imaging Kits (C10337 from Invitrogen, Carlsbad, CA, United States) was used for EdU staining according to manufacturer’s instructions. The cells were photographed using a fluorescent microscope (Olympus BX51, Tokyo, Japan), and the images were processed using Image J software (National Institutes of Health).

### Patch-Clamp Electrophysiology

Cell membrane capacitance was calculated from the time constant of a capacitance current elicited by a 5 mV depolarization from -60 mV. Pipette resistances were 2–5 mV. All current amplitudes were normalized to the cell membrane capacitance and expressed as densities (pA/pF) (Kuo et al., 2013).

### Statistical Analysis

Statistical analysis was performed using GraphPad Prism Software (version 5; San Diego, CA, United States). Data are presented as mean ± S.E.M. Differences between groups were assessed by Two-way ANOVA and Bonferroni’s test. *P* < 0.05 was considered statistically significant.

## Results

### Effects of Vildagliptin on Blood Glucose and Weight of Diabetic Mice

**FIGURE 1:**
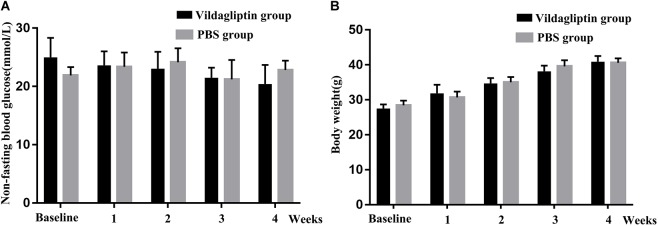
Non-fasting blood glucose **(A)** and body weight **(B)** of mice before (baseline) and after 1, 2, 3, and 4 weeks of treatment.

The average initial body weight and glucose level of the 20 mice were 28.1 ± 1.4 g, and 23.5 ± 3.1 mmol/L, respectively, which indicated that the 6-week old db/db mice could be used as diabetic mice. The mice in the two groups had comparable initial body weights and blood glucose levels. We monitored the changes in body weight and non-fasting blood glucose level in response to vildagliptin. At the end of the 4-week experiment, the body weights and blood glucose levels did not differ between the mice with or without vildagliptin treatment, although the blood glucose levels in vildagliptin treated group non-significantly decreased in a time dependent manner (Figure 1).

### Effect of Vildagliptin on Arterial Stenosis

**FIGURE 2:**
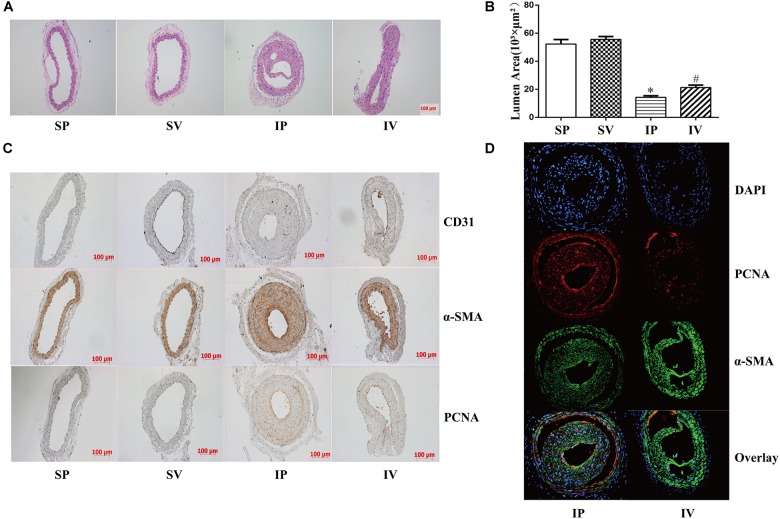
Vildagliptin inhibited ligation injury-induced neointimal hyperplasia. **(A)** Representative hematoxylin and eosin staining of carotid arteries. **(B)**. Quantifications of lumen area. ^∗^*P* < 0.05 vs. sham mice, ^#^*P* < 0.05 vs. injured mice (*n* = 3). **(C)** Representative immunohistochemical staining of α-SMA, CD31, and PCNA in carotid arteries. **(D)** Representative immunofluorescence staining of α-SMA and PCNA in injured arteries treated with or without vildagliptin.

**FIGURE 3:**
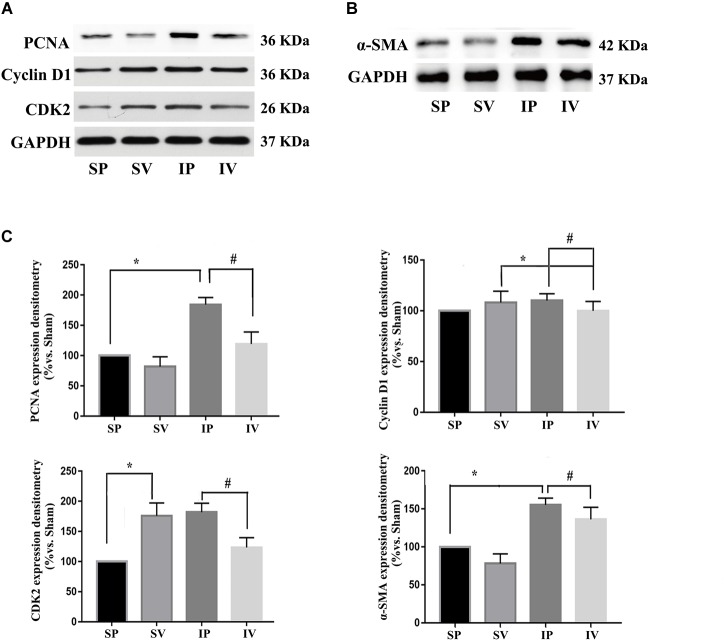
Vildagliptin inhibited ligation injury-induced proliferation and hypertrophy in VSMCs. **(A)** Representative expressions of PCNA, Cyclin D and CDK2 (indices of proliferation) analyzed by Western-blot. **(B)** Representative expression of α-SMA (an index of hypertrophy) analyzed by Western-blot. **(C)**. Quantification data of PCNA, Cyclin D1, CDK2, and α-SMA were determined by calculating the ratio of the intensity of the signal for the protein of interest to that of the normalization control. GAPDH served as the loading control. The value from the sham carotid artery treated with PBS was considered as 100% (control). Bars represented the ratio of the quantitative data from experimental groups to that from the control group. ^∗^*P* < 0.05 vs. sham, ^#^*P* < 0.05 vs. injured carotid artery (*n* = 3).

**FIGURE 4:**
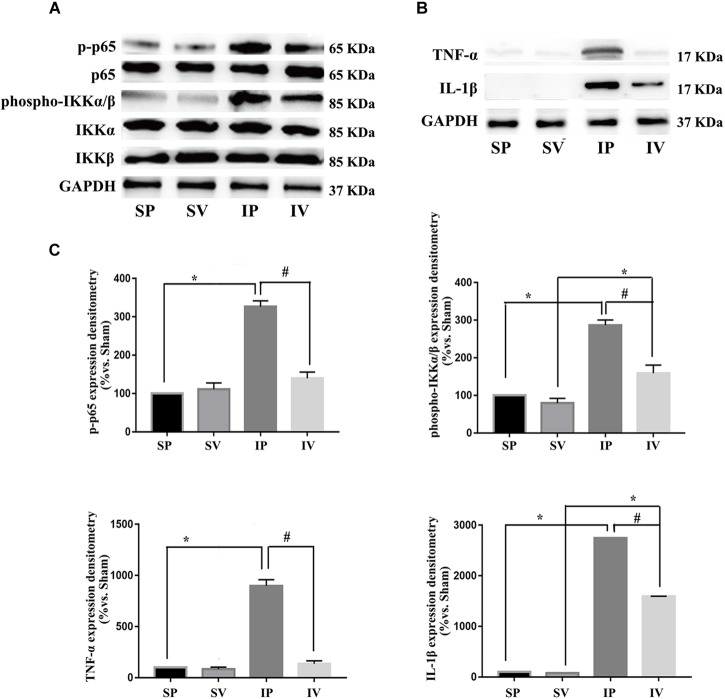
Vildagliptin inhibited arterial injury-induced activation of NF-κB pathway. **(A)** Representative expression of phospho-IKKα/β and phospho-p65 (markers of NF-κB activation) analyzed by Western-blot. **(B)** Representative expressions of TNF-α and IL-1β (downstream of NF-κB) analyzed by Western-blot. **(C)** Quantification data of p-p65 and phospho-IKKα/β were determined by calculating the ratio of the intensity of the signal for the protein of interest to that of the p65 and IKKα/β separately. Quantification data of TNF-α and IL-1β were determined by the ratio of signal for the protein of interest to that of the normalization control. GAPDH served as the loading control. The value from the sham carotid artery treated with PBS was considered as 100% (control). Bars represented the ratio of the quantitative data from experimental groups to that from the control group. ^∗^P < 0.05 vs. sham, ^#^P < 0.05 vs. injured carotid artery (*n* = 3).

**FIGURE 5:**
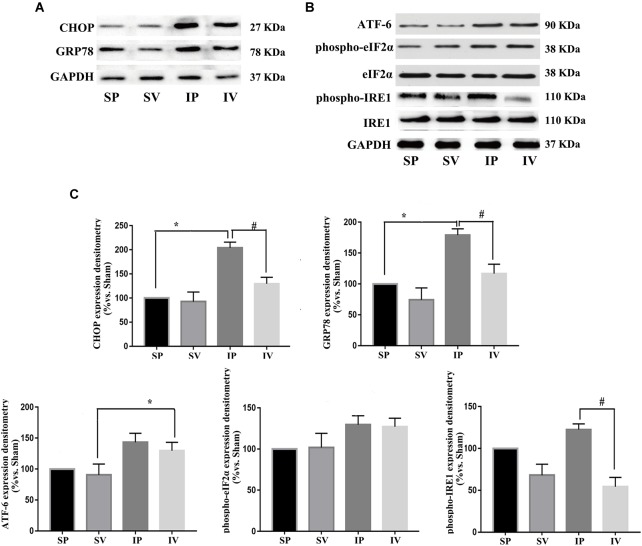
Vildagliptin inhibited ligation injury-induced activation of IRE-1 pathway. **(A)** Representative expressions of CHOP and GRP78 (markers of ER stress) analyzed by Western-blot. **(B)** Representative expressions of phospho-eIF2α, phospho-IRE1 and ATF-6 (markers of three activation pathways in ER stress, respectively) analyzed by Western-blot. **(C)** Quantification data of phospho-eIF2α and phospho-IRE1 were determined by calculating the ratio of the intensity of the signal for the protein of interest to that of the eIF2α and IRE1 separately. Quantification data of CHOP, GRP78, and ATF-6 were determined by the ratio of signal for the protein of interest to that of the normalization control. GAPDH served as the loading control. The value from the sham carotid artery treated with PBS was considered as 100% (control). Bars represented the ratio of the quantitative data from experimental groups to that from the control group. ^∗^P < 0.05 vs. sham, ^#^P < 0.05 vs. injured carotid artery (*n* = 3).

The nomenclature used for 4 different groups in Figure 2–5 is as follows: sham operations (S), injured operations (I), phosphate buffer solution treatment (P) and Vildagliptin treatment (V). The hyperplasia and hypertrophy of VSMCs are two major risk factors for restenosis subsequent to PCI. We first examined the inhibitory effect of vildagliptin on the VSMCs proliferation of injured arteries in db/db mice. After ligation injury, carotid arteries of vildagliptin treated mice showed significantly reduced intimal area and neointimal thickness compared with that of the vehicle control mice (Figure 2A,B). No difference was detected in the sham-operated arteries treated with or without vildagliptin (Figure 2A). The immunohistochemistry analysis revealed that the stenosis of injured arteries was significantly dependent on the VSMCs proliferation from the tunica media toward intima (Figure 2C). PCNA positive cells and α-SMA positive cells were significantly less in the vildagliptin treated arteries (Figure 2C). Staining of CD31 demonstrated similar endothelial coverage between control and vildagliptin treated mice (Figure 2C). Furthermore, the proliferation of VSMCs was evaluated by immunofluorescence staining of α-SMA and PCNA. As a result, the α-SMA and PCNA double-positive cells were much less in vildagliptin treated mice, indicating reduced proliferation of VSMCs (Figure 2D). These results were confirmed by the expressions of proteins associated with cell proliferation index (PCNA, cyclin D1, and CDK2) analyzed by Westernblot (Figure 3A,C). We then examined the inhibitory effect of vildagliptin on the VSMCs hypertrophy by Westernblot analysis. After ligation injury, the expression of α-SMA in carotid arteries of vildagliptin treated mice was significantly less than that in vehicle control mice (Figure 3B,C). No difference was detected in the sham-operated arteries treated with or without vildagliptin (Figure 3B,C). These results indicated that the enhanced hypertrophy was involved in stenosis subsequent to ligation injury in diabetic mice. Vildagliptin attenuated this stenosis by suppressing both proliferation and hypertrophy of the VSMCs.

### Effects of Vildagliptin on NF-κB and ER Stress Activation

After ligation injury, the expression of phospho-p65 markedly increased, accompanied by an increased expressions of phospho-IKKα/β in injured arteries (Figure 4A), indicating the activation of NF-κB pathway, which was confirmed by the expressions of TNF-α and IL-1β (the downstream of NF-κB pathway) (Figure 4B). Vildagliptin treatment inhibited the expressions of phospho-p65, phospho-IKKα/β, TNF-α and IL-1β (Figure 4A,C). In addition, after ligation injury, the expressions of GRP78 and CHOP (ER stress markers) were markedly increased, accompanied by enhanced expressions of phospho-eIF2α, phospho-IRE1 and ATF-6, which suggested that all the three pathways of ER stress were activated by the injury (Figure 5A,B). However, vildagliptin treatment only attenuated the phospho-IRE1 expression (Figure 5B,C). These results as a whole indicated that the inhibitory effects of vildagliptin on the proliferation and hypertrophy of VSMCs were achieved through inhibiting the NF-κB and IRE1 pathway in ER stress.

### Hyperglycemia Induced VSMCs Proliferation and Hypertrophy via IRE-1/NF-κB Pathway

**FIGURE 6:**
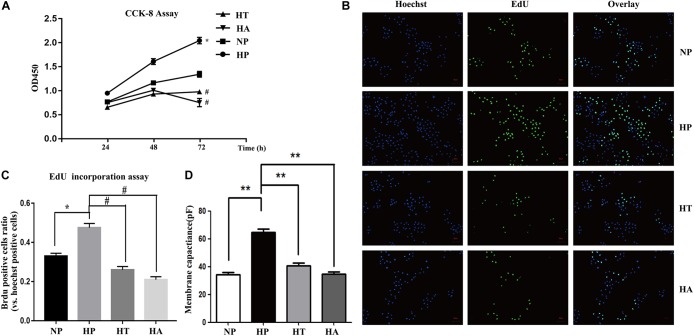
NF-κB and ER stress involved in high glucose induced proliferation and hypertrophy in VSMCs. **(A)** Enhanced proliferation in a time-dependent manner by CCK-8 assay. ^∗^P < 0.05 vs. normal glucose treated cells, ^#^P < 0.05 vs. high glucose treated cells (*n* = 5). **(B)** Representative Brdu staining of A7r5 cells in normal glucose, high glucose, high glucose treated with taurine, and high glucose treated with ACHP by EdU incorporation assay. **(C)** Quantifications of Brdu positive cells to hoechst positive cells ratio (*n* = 5). **(D)** The mean values of cell capacitances in cells in normal glucose, high glucose, high glucose treated with taurine, and high glucose treated with ACHP conditions. ^∗∗^P < 0.05 (*n* = 5).

**FIGURE 7:**
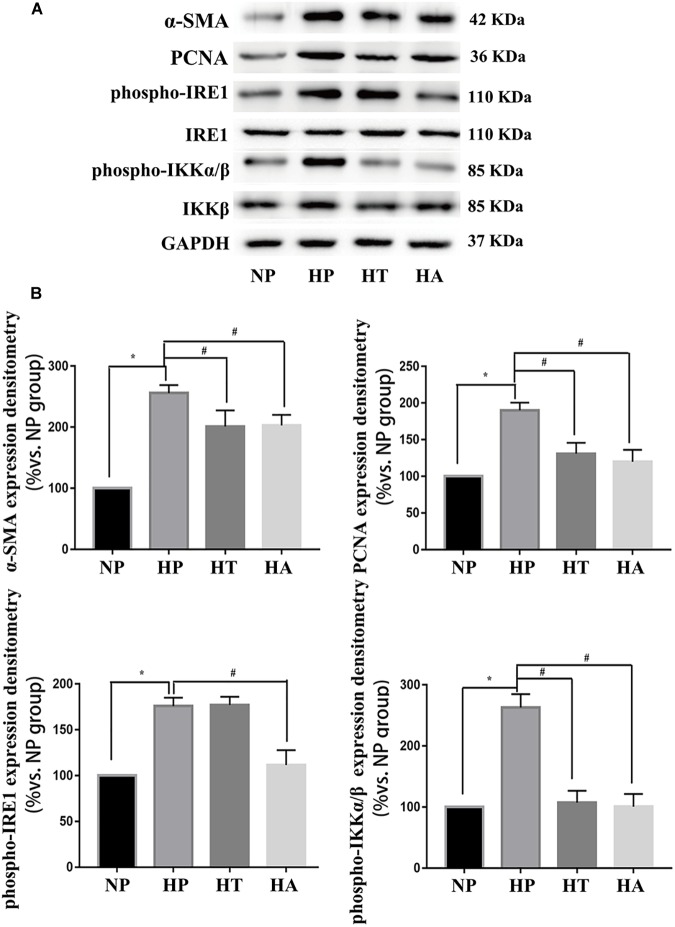
IRE-1 pathway involved in high glucose induced NF-κB activation. **(A)** Representative expressions of PCNA (an index of proliferation), α-SMA (an index of hypertrophy), phospho-IRE1 (marker of activation in ER stress IRE-1 pathway), phospho-p65 (marker of NF-κB activation) in A7r5 cells in normal glucose, high glucose, high glucose treated with taurine, and high glucose treated with ACHP conditions by Western-blot assay. **(B)** Quantification data of phospho-IRE1 and phospho-IKKα/β were determined by calculating the ratio of the intensity of the signal for the protein of interest to that of the IRE1 and IKKβ separately. Quantification data of α-SMA and PCNA were determined by the ratio of signal for the protein of interest to that of the normalization control. GAPDH served as the loading control. The value from the normal glucose treated cells was considered as 100% (control). Bars represented the ratio of the quantitative data from experimental groups to that from the control group. ^∗^P < 0.05 vs. normal glucose treated cells, ^#^P < 0.05 vs. high glucose treated cells (*n* = 3).

The nomenclature used for 4 different groups in Figure 6, 7 is as follows: high glucose treatment (H), normal glucose treatment (N), phosphate buffer solution treatment (P), taurine treatment (T) and ACHP treatment (A). To verify the role of NF-κB and ER stress in the proliferation and hypertrophy of VSMCs in hyperglycemia condition, we treated A7r5 cells in 33 mmol/L glucose with or without the ACHP (pharmacological inhibitor of IKK) and taurine (pharmacological inhibitor of IRE-1). The CCK-8 and EdU incorporation assay were used to determine the proliferation of A7r5 cells, while the hypertrophic state of A7r5 cells was determined by cell capacitance using whole cell voltage patch-clamp technique. As shown in Figure 6, the proliferation was significantly more remarkable and the capacitance in A7r5 was significantly higher after treated with high concentration of glucose than that treated with normal concentration of glucose, and pretreatment with ACHP or taurine inhibited these effects. On the other hand, it were observed that both the expressions of PCNA and α-SMA, and the expressions of phospho-IKKα/β and phospho-IRE-1 significantly increased after the cells being treated with high glucose, and all these effects could be reversed by pretreatment with ACHP or taurine (Figure 7A), which suggested that high glucose treatment could induce the proliferation and hypertrophy of VSMCs via NF-κB and IRE-1 pathway. In cells treated with both high glucose and taurine, phospho-IRE-1 and phospho-IKKα/β expressions significantly decreased compare to those treated with high glucose alone (Figure 7A). However, ACHP increased the expressions of phospho-IKKα/β, but did not affect the activation of IRE-1, which suggested that IKK was downstream from IRE-1 activation (Figure 7A,B). In summary, the results suggested that IRE-1/NF-κB pathway was involved in hyperglycemia induced proliferation and hypertrophy of VSMCs.

## Discussion

Our study demonstrated for the first time that vildagliptin treatment reduced the stenosis of ligation injured carotid artery in diabetic mice, and this effect was not associated with the glycaemia or body weight. We further proved that the anti-proliferative and anti-hypertrophic effects of vildagliptin were achieved through inhibiting VSMCs proliferation via ER stress/NF-κB pathway.

It is known that hyperglycemia makes a major contribution to the activation of ER stress (Gorman et al., 2012; Jager et al., 2012). In our study, a 4-week administration of vildagliptin significantly decreased the expressions of GRP78, CHOP, and phospho-IRE-1/IRE-1 in carotid arteries of the diabetic mice, in which GRP78 and CHOP are regarded as two important markers of the activation of ER stress (GRP78 is a molecular chaperone of ER. CHOP is activated under ER stress and serves as an apoptotic modulator) (Werstuck et al., 2001), and IRE-1 is one of the three main signal pathways involved in the activation of ER stress (Szegezdi et al., 2006; Hetz et al., 2013). Our results indicated that in the diabetic mouse model, ER stress responses to hyperglycemia were attenuated by vildagliptin.

It was reported that Vildagliptin augmented plasma active GLP-1 concentrations and prolonged the action of GLP-1 in db/db mice (Wu et al., 2015). The enhanced GLP-1 exhibited a number of salutary vascular actions via binding to GLP-1 receptors expressed on VSMCs (Goto et al., 2011). Shimoda et al. (2011) found that the expression of ER stress markers (ATF4 and PERK) could be down-regulated by the activation of GLP-1 receptors in pancreatic beta cells. Similarly, we found that the indexes of ER stress (the expression of CHOP, GRP78, and phospho-IRE1) were reduced in the VSMCs after Vildagliptin treatment, of which the phospho-IRE1 could regulate the activation and translocation of NF-kB (Zha et al., 2015; Wang N. et al., 2017; Chen et al., 2018). Accordingly we determined the expressions of phospho-IKKα/β and phospho-p65 in VSMCs, and the results revealed that vildagliptin administration down-regulated the activation of NF-κB, which was also proved by the decreased expressions of both TNF-α and IL-1 (Miyagawa et al., 2013). Taking together, our results suggest that vildagliptin attenuated the *in vivo* NF-κB responses via IER-1/IKK pathway in VSMCs. Additionally, phosphorylation levels of IKK-α/β decreased in taurine or ACHP treated VSMCs (A7r5), while phosphorylation levels of IRE-1 decreased only in taurine treated VSMCs *in vitro*, which suggest that ER stress responses and its down-stream NF-κB activation were attenuated by vildagliptin. These results would confirm that vildagliptin administration inhibits the occurrence and development of inflammation.

Previous studies have indicated that NF-κB modulates the expressions of genes involved in cellular and physiological process, such as proliferation and migration (Nabata et al., 1990; Bellas et al., 1995). In our study, expressions of cyclinD1 and cyclin-dependent kinase 2 were all significantly inhibited by vildagliptin administration in consistent with the down-regulation of NF-κB responses, indicating that transcriptional activation of G1 phase Cyclins and subsequent G1/S phase transition were restrained. Additionally, the hypertrophic state of VSMCs induced by hyperglycemia was reversed after blocking NF-κB signaling pathway with ACHP. These results suggested that down-regulation of NF-κB played a key role in suppressing the proliferation and hypertrophy processes of VSMCs by vildagliptin.

It is worth to note that the effect of vildagliptin is completely different in islets cells. Emerging evidence reported that vildagliptin exert pro-survival and proliferative effect through increasing pancreatic insulin (Wu et al., 2015). The different responses between tissues may owe to the different functions of ER stress related proteins. In pancreatic islets, vildagliptin protects islet beta cell from death by reducing expression of ATF-4, which contributes to the cell apoptosis (Yusta et al., 2006). Conversely, vildagliptin reduces the expression of phospho-IRE-1 in VSMCs, and consequently decreases the activation of NF-κB, which is associated with the inflammation and proliferation (Hotamisligil and Erbay, 2008; Verfaillie et al., 2013).

Our study has potential limitations. First, we aimed to investigate the level of the NF-κB response of the VSMCs in the carotid artery, so we just measured the expressions of TNF-α and IL-1β of the VSMCs, but not the circulating levels of TNFa and IL-1β. Anyway, a study reported by Miyagawa et al. (2013) showed that the plasma levels of TNF-α and IL-6 were significantly decreased after 8-week vildagliptin treatment in db/db mice, which could be served as a reference. Second, as Vildagliptin would not directly act on the VSMCs *in vitro* due to lack of the GLP-1/GLP-1 receptors (Shi et al., 2015), so we had to use the pharmacological inhibitors, taurine and ACHP, to simulate the functions of Vildagliptin on VSMCs while investigating the mechanism of the anti-proliferative and anti-hypertrophic effects of Vildagliptin *in vitro*. As a result, after suppressing ER stress and NF-κB responses, the proliferation and hypertrophy of A7r5 cells were significantly inhibited. We believe these would imitate the same mechanism of the anti-proliferative and anti- hypertrophic effects of Vildagliptin on VSMCs *in vivo*.

## Conclusion

Our study indicates that excessive proliferation of VSMCs plays a dominant role in the pathogenesis of stenosis induced by ligation injury. Vildagliptin inhibits proliferation and hypertrophy of VSMCs via regulating the expression of ER stress and its downstream NF-κB pathway. These findings may lead to a novel therapeutic strategy for treating restenosis in diabetic patients.

## Author Contributions

TY, YG, and CL designed the experiments. YJ, YF, and JZ carried out experiments. SY, LM, XZ, and LY analyzed the data. YJ, YG and CL wrote the manuscript. All authors read and approved the final version of manuscript.

## Conflict of Interest Statement

TY received a grant of “DPP-4 Science Award Program of China” from Novartis Pharmaceuticals (China). The remaining authors declare that the research was conducted in the absence of any commercial or financial relationships that could be construed as a potential conflict of interest.

## Supplementary Material

TABLE S1 Original data of Figures 1–7.
